# Effectiveness of Individual Feedback and Coaching on Shared Decision-making Consultations in Oncology Care: Protocol for a Randomized Clinical Trial

**DOI:** 10.2196/35543

**Published:** 2022-04-06

**Authors:** Haske van Veenendaal, Loes J Peters, Dirk T Ubbink, Fabienne E Stubenrouch, Anne M Stiggelbout, Paul LP Brand, Gerard Vreugdenhil, Carina GJM Hilders

**Affiliations:** 1 Erasmus School of Health Policy and Management Erasmus University Rotterdam Rotterdam Netherlands; 2 Dutch Association of Oncology Patient Organizations Utrecht Netherlands; 3 Surgery Amsterdam UMC location University of Amsterdam Amsterdam Netherlands; 4 Medical Decision Making, Department of Biomedical Data Sciences Leiden University Medical Centre Leiden Netherlands; 5 Department of Innovation and Research Isala Hospital Zwolle Netherlands; 6 Department of Oncology Máxima Medical Center Eindhoven Netherlands; 7 Board of Directors Reinier de Graaf Hospital Delft Netherlands

**Keywords:** decision-making, shared, education, professional, feedback learning, coaching, medical consultation, medical oncology, palliative care

## Abstract

**Background:**

Shared decision-making (SDM) is particularly important in oncology as many treatments involve serious side effects, and treatment decisions involve a trade-off between benefits and risks. However, the implementation of SDM in oncology care is challenging, and clinicians state that it is difficult to apply SDM in their actual workplace. Training clinicians is known to be an effective means of improving SDM but is considered time consuming.

**Objective:**

This study aims to address the effectiveness of an individual SDM training program using the concept of deliberate practice.

**Methods:**

This multicenter, single-blinded randomized clinical trial will be performed at 12 Dutch hospitals. Clinicians involved in decisions with oncology patients will be invited to participate in the study and allocated to the control or intervention group. All clinicians will record 3 decision-making processes with 3 different oncology patients. Clinicians in the intervention group will receive the following SDM intervention: completing e-learning, reflecting on feedback reports, performing a self-assessment and defining 1 to 3 personal learning questions, and participating in face-to-face coaching. Clinicians in the control group will not receive the SDM intervention until the end of the study. The primary outcome will be the extent to which clinicians involve their patients in the decision-making process, as scored using the Observing Patient Involvement–5 instrument. As secondary outcomes, patients will rate their perceived involvement in decision-making, and the duration of the consultations will be registered. All participating clinicians and their patients will receive information about the study and complete an informed consent form beforehand.

**Results:**

This trial was retrospectively registered on August 03, 2021. Approval for the study was obtained from the ethical review board (medical research ethics committee Delft and Leiden, the Netherlands [N20.170]). Recruitment and data collection procedures are ongoing and are expected to be completed by July 2022; we plan to complete data analyses by December 2022. As of February 2022, a total of 12 hospitals have been recruited to participate in the study, and 30 clinicians have started the SDM training program.

**Conclusions:**

This theory-based and blended approach will increase our knowledge of effective and feasible training methods for clinicians in the field of SDM. The intervention will be tailored to the context of individual clinicians and will target the knowledge, attitude, and skills of clinicians. The patients will also be involved in the design and implementation of the study.

**Trial Registration:**

Netherlands Trial Registry NL9647; https://www.trialregister.nl/trial/9647

**International Registered Report Identifier (IRRID):**

DERR1-10.2196/35543

## Introduction

### Background

Shared decision-making (SDM) has been promoted to support patients in making informed decisions that best fit their personal preferences, circumstances, and concerns [1,2]. This is particularly important in oncology as many treatments involve serious side effects, and treatment decisions involve a trade-off between benefits and risks [3,4]. Approximately 110,000 Dutch patients are diagnosed with cancer each year [5]. Surgery, radiation, and systemic treatment options are available for most patients with cancer. The made treatment decisions determine crucial aspects of the lives of all patients and their families. Being diagnosed with cancer brings emotional stress, which affects patients’ information recall and the decision-making process [6-8].

However, SDM implementation in oncology is challenging [9-12]. There is a relatively high level of uncertainty in cancer care regarding the treatment benefits and risks [10,12,13]. *Fighting* cancer is paramount in the focus of both clinicians and patients, which may impede the process of considering multiple treatment options and weighing their short- and long-term consequences [14-16]. Moreover, different clinicians within a team must coordinate the decision-making process over an extended period and for several decisions, which makes it difficult to guarantee continuity in the decision-making process [4]. Interventions tailored to specific local contexts have been proposed to stimulate the integration of SDM in usual care [17-21].

In addition, clinicians underline the importance of communication with their patients but feel that it is difficult to apply SDM in their actual workplace and believe that applying SDM does not differ much from their current practice [22-24]. Training clinicians as part of the implementation of SDM is generally seen as vital to overcome these hurdles [22,25-29]. Training involves theory and skills but is more effective when it also accounts for peer pressure, individual attitudes, and learning objectives [30]. It has been suggested that elements such as reflection and real time feedback be added to a clinician’s actual SDM performance [31]. Recent efforts that incorporate feedback from observations of consultations to improve SDM competencies are promising [23,29,32].

SDM behavior is complex as it comprises interacting elements that are also influenced by contextual factors [32-34]. Medical professionals are expected to continuously improve their knowledge, skills, and behaviors, which requires the development and use of reflective practice skills [35,36]. Regarding medical performance, it has been stated that additional experience will not improve once it reaches the level of automaticity and effortless execution [37]. Deliberate practice involves the provision of immediate feedback, time for problem-solving and evaluation, and opportunities for repeated performance to refine behavior [37,38]. As deliberate practice supports teaching that is more focused on the motivation and self-directed learning of the clinician, coaching is being increasingly recognized as a method of enhancing technical and nontechnical clinical performance [39-42]. Effective coaching on complex communication skills, including those involved in SDM, requires direct observation or review of audio- or video-recorded health care encounters, followed by constructive feedback from the coach and the processing of this feedback into developmental actions by the coachee [43,44]. As training clinicians—face to face, individually, or in a team—is time consuming and challenging for a busy health care team [26,45], training approaches that improve SDM behaviors should be both effective and feasible. The effects of deliberate practice have not been evaluated in the design of effective SDM education but coincide with clinicians’ own views that feedback and reflection, tailored to their own learning needs and firmly embedded in the daily working context, are considered vital to effectively learn communication skills [46].

### Objective

The aim of this randomized clinical trial is to examine whether an individual SDM training program for oncology clinicians grounded in the theory of deliberate practice [37], as compared with their standard clinical practice, improves SDM behavior. The program comprises audiotaping the consultation or consultations of a single patient and conducting an SDM e-learning program containing both theory and a role-play example, followed by self-assessments, individual feedback reports, and coaching facilitated by an individual action-planning template.

## Methods

### Trial Design

This multicenter, single-blinded randomized clinical trial was designed and will be reported in accordance with the CONSORT (Consolidated Standards of Reporting Trials) guidelines [47]. The trial addresses the effect of SDM interventions in real-life clinician-patient consultations on the extent to which clinicians involve their patients in the decision-making process. The design is unpaired, meaning that patients are only audiotaped once, either before or after the intervention. In the control group, the clinicians will not receive the SDM intervention until the trial period has finished. The trial will include different oncology clinicians, diagnoses, hospitals, and decisions to investigate applications in a range of oncological diagnoses, including patients in palliative care.

### Study Conduct

When joining the study, clinicians will complete a short questionnaire asking about their number of years of experience, former participation in SDM skills training (yes or no) during medical school or as part of continuous medical education, residency, profession, age, and gender. The diagnosis, gender, and age of the patients will be recorded by the clinician to gather the basic demographic data of the study sample.

A measurement involves recording ≥1 consultation relevant to a decision-making process of 1 patient only, with a questionnaire that measures patients’ perceived involvement in the decision-making process. The physicians and patients will be aware that consultations are being recorded. Each clinician will record the decision-making process for 3 different patients. By recording 2 consultations after the SDM intervention, with a time interval of 3 to 4 weeks between the recordings, the effectiveness of the SDM intervention for clinicians can be measured over time. The duration of the consultations and coaching sessions will be noted by the researcher (HvV) directly from the recordings. Clinicians will be instructed not to participate in educational activities related to patient-centered communication during the study. In addition, clinicians in the intervention group will be asked not to discuss the training contents or study-related information with participants in the control group. Once the final consultation is recorded, clinicians in the control group will receive the equivalent communication training. The period between each measurement will be 3 to 4 weeks, summing up to a total participation of approximately 8 weeks per clinician.

### Participants

A total of 12 hospitals in the Netherlands will be included in this study (n=3, 25% universities; n=5, 42% general teaching; and n=4, 33% district hospitals). The recruitment of consecutive clinicians, who will discuss treatment decisions with their patients, will take place from April 2021 to July 2022.

All clinicians from the 12 hospitals involved in the decision-making process with patients of oncology regarding treatments will be invited to participate in the study. Clinicians in training (residents) are also eligible as, in the Dutch situation, they work under supervision but communicate with patients independently. Clinicians who have already received individual feedback on consultations or participated in SDM training within the past 3 years will be excluded. The inclusion criterion is that clinicians should be conducting consultations in which a decision is to be made with a patient who is capable and willing to participate. In addition, choices may relate not only directly to the final treatment decisions but also to other aspects of the care process. Consultations with patients who are palliatively treated with no prospect of cure, for whom decisions are to be made regarding the quality of life, are also eligible.

### Intervention

#### Overview

To clarify what SDM entails when applied in daily practice, we will invite clinicians to reflect on their own communication behavior during ≥1 consultation in which a treatment decision is made in relation to the following four steps for applying SDM: (1) creating option awareness, (2) discussing the options and their pros and cons, (3) exploring patients’ values, and (4) agreeing on a decision that fits best with the patients’ personal preferences [48]. All participants receive a *crib sheet*, a pocket-sized card to be used during or in between consultations that shows the 4 SDM steps with example phrases. These 4 steps are also key elements in the educational components of our intervention.

To support the adoption of SDM behavior by clinicians in daily practice, we will use the following four implementation levels of the Meetinstrument Determinanten van Innovaties model and their change determinants for our implementation approach [21]: (1) innovation (the implementation of SDM), (2) users of the innovation (clinicians and patients), (3) organizational context, and (4) sociopolitical context. To take the social context into account, oncology clinicians will be asked to participate as teams to enhance implementation success. By asking for a fee for participation in the training, we also ensure financial commitment from the hospitals to increase legitimacy and adherence to the trial.

Next, we will use the principles of deliberate practice as the basis for the educational approach. The best training situations focus on activities of short duration with opportunities for immediate feedback, reflection, and corrections [37]. In addition, additional reinforcing principles of medical coaching and action learning have been added [49-55].

The full SDM intervention takes <2.5 hours and comprises 4 parts, as described in the following sections.

#### e-Learning (45 Minutes)

An e-learning program was developed to comprehensively explain the principles and theoretical background of SDM. It addresses knowledge (ie, deﬁnition, rationale, effect, and the 4 steps for applying SDM); attitude (ie, reported barriers, own beliefs, and providing evidence on frequent misconceptions about SDM) [52]; and, to a lesser extent, self-efficacy illustrated with a video example of a consultation following the 4 steps of SDM. In e-learning, information is given about patients’ perspectives on SDM based on internet polls among (former) patients. A total of 7 questions will be asked during the 45-minute e-learning program to stimulate reflection and memory. e-Learning was used and evaluated in a former implementation project on breast cancer [23,32]. The completion of basic SDM e-learning will be mandatory. Additional e-learning may be completed on a voluntary basis.

#### Reflection on Feedback Report (15 Minutes)

Participants will receive a personal feedback report from a communication researcher based on the Observing Patient Involvement–5 (OPTION-5) scores of their own consultation or consultations recording of a decision process with a patient [30]. This individual report will contain a score (0-4) per OPTION-5 item, as well as illustrative quotes and behaviors during the encounter that contributed most to a score and comprises 1 to 2 pages of ≥1 consultation per patient. The report was tested in 11 teams comprising patients with breast cancer during former implementation projects [23,32]. The direct observation of clinical encounters followed by structured feedback and coaching is educationally valuable [30] and seems promising for improving SDM behaviors [29,56,57]. By recording an actual clinical consultation in which a decision with a patient is made, feedback can be provided, and the recording can be stopped at critical points to reflect on and discuss appropriate goals with the coach. We put emphasis on quotes and nonjudgmental feedback rather than using a summative assessment form, as clinicians might feel this may reduce communication skills to behavioral components and may perceive this as impeding the improvement of their communication skills [46].

#### Self-assessment and Defining 1 to 3 Personal Learning Questions (30 to 45 Minutes)

This feedback will be aligned with the learner’s ambition by giving clinicians a short version of the OPTION-5 checklist to complete a self-assessment of their recording. Next, we strive to provide feedback as individualized as possible and as close to their clinical reality by using quotes and linking the quotes to a practical 4-step model that can be used in the consultation. In addition, clinicians will then be asked to write down 1 to 3 learning questions, which will help reflect on their own performance. In addition, defining a personal ambition stimulates intrinsic behavioral changes. Participants will use e-learning, self-assessment, and personal feedback reports to reflect on what would help them improve the adoption of SDM in their daily practice the most. Writing down learning questions is the first part of the action-planning template, which is provided to serve as a checklist for the coaching session, self-reflection, and follow-up of planned actions.

#### Face-to-face Coaching: 15 to 30 Minutes

**Figure 1:**
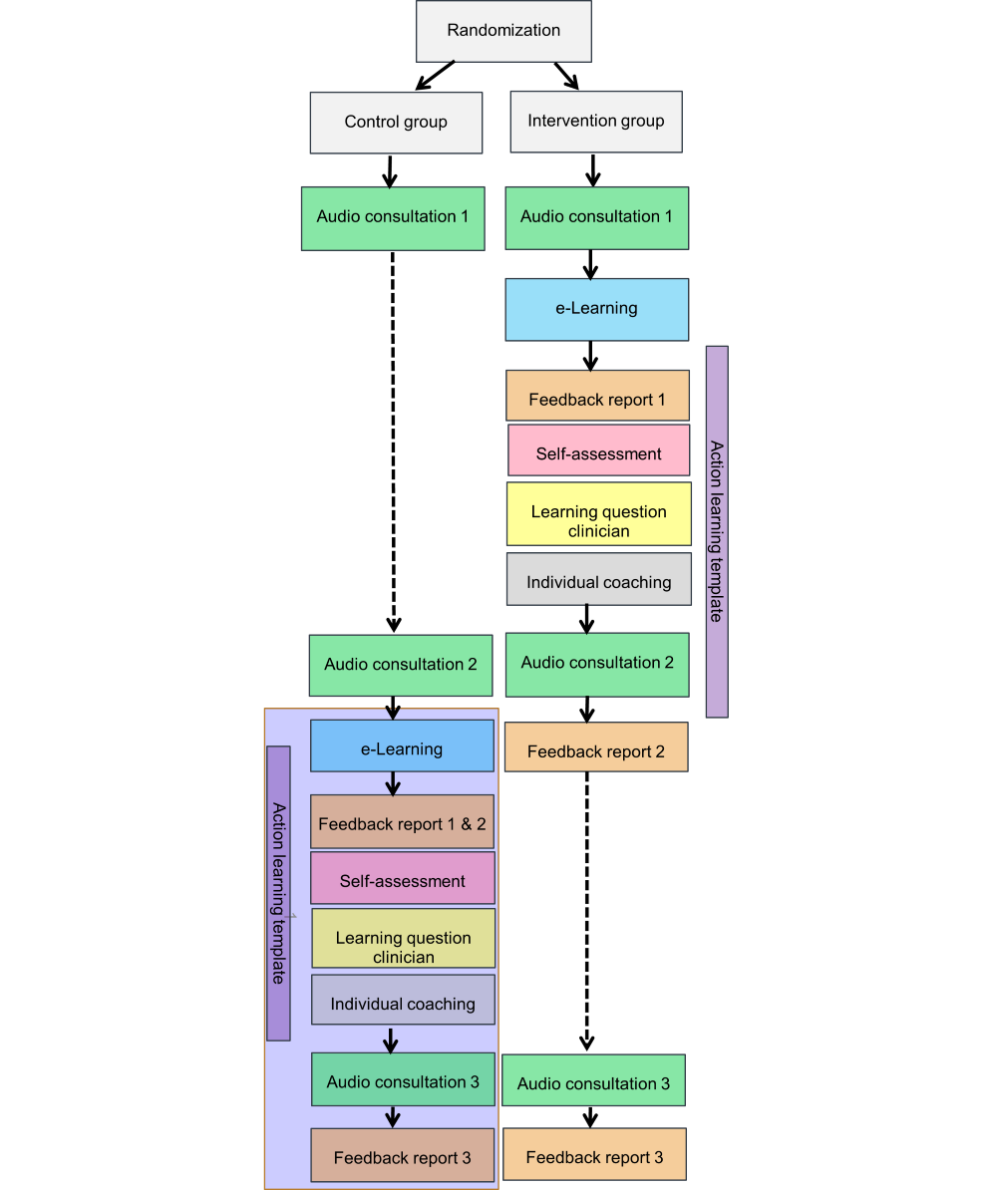
Design of the study.

Clinicians will discuss the feedback with an experienced communication coach (HvV, Maaike Schuurman, or Esther van Weele) using both the participants’ learning question or questions and the feedback report. To support reflexive and action learning, all participants will be provided with an action-planning template [50]. A model for effective coaching [40] will be used that involves four steps: (1) establishing principles of the relationship, (2) conducting an assessment, (3) developing and implementing an action plan, and (4) assessing the results of action plans and revising them accordingly. After the coaching session, each clinician will complete the action-planning template to force them to reflect on their SDM behavior, consider goals, and decide which strategies and skills will help them attain those goals. The coaching model is explained in Table 1, and the study design is presented in Figure 1. A professor of clinical medical education (PB) was consulted to finalize the form of coaching. In addition, an evaluation of the coaching will take place after 3 and 10 coaching sessions. After the coaching, the following characteristics of the coaching session will be noted: the content of the session; action planning; duration of the session; whether the clinicians prepared the learning objectives, relistened their own consultation, and read the feedback report beforehand; and the number of e-learnings completed.

**Table 1:** Elements and working constructs of effective coaching.

Element for effective coaching [40]	Working construct	Translation to our coaching approach [39,43,53,54]
Establishing principles of the relationship	Establish goals and parameters of the relationship, as well as ethical considerations, including confidentiality and boundary issues	Express roles: the learner sets goals and designs the actions that help to apply SDM^a^; the coach makes suggestions and encourages the learner to define actions to realize ambitions Downplay the coaches’ role: position the coach as a learner, not an expert, to establish a nonhierarchical relationship that contributes to creating a safe space, as well as to coconstruct meaning and knowledge rather than to dictate it; emphasize that interdependence is the basis of valuable interaction Facilitate honest discussion about strengths and challenges regarding SDM; help clinicians shift their focus from performance to learning Make room for discussing areas for improvement of applying SDM in daily practice Ask about the positive consequences the learner expects to accomplish with applying new SDM behavior
Conducting an assessment: self-assessment and assessment by a communication coach	To facilitate a feedback process to begin self-monitoring and encourage learners to gain reflective skills to help them set goals for their program through personal (to foster discovering the students’ learning or interpersonal management style) and systemic assessments (assessments provided by the learner’s program)	In general, active and appreciative listening and asking questions; stimulate reflection: capable of being introspective and learning from yourself Ask about the importance of SDM for the learners’ professional role and development Provide written feedback, after permission, of audio-recorded consultation or consultations of the learner with a patient in which a decision is made A self-assessment is performed by listening back to their own consultation and using a shortened OPTION-5^b^ measurement tool Ask the learner to draw up 1 to 3 personal learning questions for the coaching session based on personal ambition and feedback Review the written feedback that was provided together and whether it was recognizable to promote self-reflection and goal setting as the foundation of self-regulated learning [43] Discuss the theory of SDM: what does it intend? What insights and questions come from the e-learning? Use the 4-step model as a mirror for reflection on feedback and the goals Use practical examples from best practices, including prompts, of potential areas of struggle to help learners identify challenges
Developing and implementing an action plan	This step determines new and revised actions that will lead to goal attainment; the learner reflects on what is working and what is not working, relate these to their learning style, and identifies learning opportunities that build knowledge and skills or initiates actions that demonstrate the learner’s progress toward competence	Focus discussion to areas of dilemmas and best cases to create action ideas; ask the learner what they need to accomplish their expressed ambitions regarding SDM If clinicians express the wish to gain knowledge about SDM (ie, evidence for the use of teach-back, decision aids, background information about SDM measurement tools, or theory about elicitation values and preferences), we will provide handy cards, decision tools, support (ie, decision tools and tips to apply SDM as a team), or written information to read Facilitate the transition from self-assessment and feedback to intervention: collaboratively crafting an action plan to implement appropriate intervention strategies [50,51] Encourage the learner set 1 to 3 goals to be attempted in the next consultation and establish a short action-planning template Ask questions to make goals ISMART^c^ Ask the learner about possible barriers to or facilitators of achieving their expressed goals and discuss possible ways of coping with them to increase clinicians’ level of confidence in achieving the planned actions and coping with the feelings of failure
Assessing the results of action plans and revising accordingly	The coach and the learner review and evaluate the learners’ progression according to the action plan and whether features of the plan should be revised	The action-planning template ends with identifying at least two goals for their clinical practice over the ensuing weeks After the coaching session, clinicians will receive feedback on their aspired goals, integrated as part of the feedback on their consultation Evaluate the session and ask if there are any issues left to discuss If a next meeting is desired, plan the date and agenda for the next meeting Finally, residents will complete a brief evaluation, with Likert scale response options, that addresses the acceptability and usefulness of coaching

^a^SDM: shared decision-making.

^b^OPTION-5: Observing Patient Involvement–5.

^c^ISMART: important, specific, measurable, accountable, realistic, and timeline.

### Comparator

The time schedule for participants randomized to the control group is shown in Figure 1. They will first be asked to complete the recording of the decision-making process of 2 different patients before they are offered the intervention (including recording a third decision-making process). This will enable a comparison of their SDM behavior with participants who are exposed to the intervention. By offering the intervention to participants in the control group after the trial period, we will ensure that all participants in this trial have the opportunity to develop themselves in the field of SDM. To keep similar trial circumstances, the interval between these 3 recordings (3-4 weeks) will be similar to that of the intervention group.

### Outcomes

The primary outcome is the OPTION-5 instrument to rate the clinicians’ behavior in the decision-making process objectively, which will be performed by 2 of the 3 researchers (HvV and Maaike Schuurman and Esther van Weele) independently [30]. Each of the 5 items will be rated on a scale ranging from 0 (no effort made) to 4 (exemplary effort made).

As secondary outcomes, we will use subjective measures of SDM scored by the patients: the iSHARE, Control Preferences Scale (CPS), and the SDM Questionnaire–9 (SDM-Q-9) questionnaires.

The 15-item iSHARE questionnaire measures the perceived level of SDM during medical consultation or consultations; it was recently developed and has shown adequate content validity and comprehensibility [55]. It covers the entire SDM process rather than a single consultation and involves both clinician and patient behaviors. It is especially meant for the oncology setting, as definitions of SDM differ between health care settings [58]. The CPS has proven to be a clinically relevant, easily administered, valid, and reliable measure of preferred or experienced roles in decision-making among people with life-threatening illnesses [59]. The CPS comprises 1 question with 5 possible statements indicating the role of the clinician and patient in the decision-making process. The SDM-Q-9 comprises 9 statements. For each statement, patients rate the extent to which they *completely disagree* (0) to *completely agree* (5) on a 6-point Likert-type scale. The scores are added, multiplied by 20, and divided by 9 to provide a percentage of the maximum score, ranging from 0 (no SDM) to 100 (maximum level of SDM). If needed, a maximum of 2 missing items will be imputed with the mean of the items that are scored [60]. The duration and number of consultations are registered for each physician directly from the audiotaped consultation or consultations.

### Sample Size

The primary outcome of this trial will be the extent to which clinicians involve their patients in the decision-making process, as scored using the OPTION-5 instrument [30,61]. A ≥10-point improvement in the OPTION-5 score is considered clinically relevant and significant, given the relatively limited time investment of the participants. For instance, a >10-point OPTION-5 score indicates 2 out of 5 items improving from moderate effort (2 points) to skilled effort (3 points) or 1 item improving from minimal effort (1 point) to skilled effort (3 points).

A preintervention mean score of 38 is assumed for our sample, which was measured in a former implementation project involving 6 outpatient breast cancer teams [32]. This is a high baseline score compared with other studies in general [56] and for oncology [9,11,57]. A total sample size in a 2-sided *Z* test for 2 means of 72 patients will be calculated based on an increase in the OPTION-5 score from 38 before implementation to 48 after implementation, with an SD of 13 in both groups, achieving a 90.38% power at the 5% significance level [13,32,56]. We will expand the sample size to 100 clinicians to account for possible failed recordings and dropouts of clinicians. A subanalysis will be performed to evaluate whether the results for palliative decisions, that is, patients who are palliatively treated (both tumor targeted and non–tumor targeted), are similar to those for the group with curative treatment intentions.

### Randomization and Blinding

Randomization (Figure 1) will be conducted by allocating each clinician agreeing to participate in the study to either the intervention arm or the control arm (1:1) based on randomly mixed block sizes (2, 4, or 6) using Castor EDC (Castor Company) [62]. This type of randomization is common in multicenter studies that include approximately 100 participants to reduce the predictability of allocation [63]. All patients and raters will be blinded, whereas clinicians cannot be blinded to their allocation. The allocation sequence, enrollment, and assignment of participants to interventions will be conducted by a coordinator (LP) not involved in rating consultations and coaching of the participants.

### Statistical Methods

All raters will use the OPTION-5 coding scheme, which has been refined for patients of oncology and vascular surgery [61,64]. The manual will be adjusted to be relevant to the oncology setting to increase raters’ agreement in scoring the audio recordings. All audio recordings will be scored independently by 2 raters blinded to the intervention using the OPTION-5 instrument. After the first 10 audio recordings, these scores will be compared, and the coding rules will be discussed to reach an agreement over the final score. Moreover, the personal feedback and coaching sessions with the first 10 clinicians will be discussed by the project team in which patients are involved, and the unweighted Cohen κ values will be calculated as a measure of the interrater agreement [65]. The OPTION-5 score will be converted from a 0- to 20-point scale into a 0% to 100% scale.

Descriptive statistics will be presented as percentages or means with SDs. Differences will be expressed as mean differences with 95% CIs. The Pearson chi-square statistic will be used to analyze the differences between categorical variables at *P*<.05. We will check whether previous training in communication skills, professional background, disease, duration of the consultation or consultations, hospital, age, and number of consultations are equally distributed between the study arms. If they are not equally distributed, they will be included in the regression model for the OPTION-5 score. We will also perform a subanalysis for palliative decisions to evaluate whether the effectiveness of the SDM intervention for these consultations is comparable with that for the entire group. Statistical analyses will be performed using SPSS Statistics (version 25; IBM Corporation).

### Patient Involvement

To guarantee that the patient’s perspective is sufficiently included in the design of the SDM intervention, 2 patient representatives (Maaike Schuurman and Ella Visserman) and 1 (former) patient with breast cancer (Lisanne de Groot) have been involved in the study. The 2 patient representatives have been involved from the start of setting up the research project (including determining research questions and outcome measures) as part of the research team in recruiting clinicians for the study and are also committed to disseminating the study results and methodology in oncology care. A patient representative (Maaike Schuurman) is involved as a researcher in rating consultations with the OPTION-5 instrument and providing coaching to clinicians (Maaike Schuurman), and all three (Maaike Schuurman, Ella Visserman, and Lisanne de Groot) will give feedback on specific parts of the training program, such as the content of the coaching sessions and feedback reports.

### Ethics Approval and Informed Consent

All participating clinicians will receive information about the study and will be asked to give verbal consent for participation in the study: providing contact details, selecting a patient, and recording a consultation will be considered as their verbal consent. Their patients will complete a written consent form as consultations will be audio recorded, and patient characteristics will be collected. Non–Dutch-speaking patients will be excluded unless they are accompanied by a person who speaks Dutch sufficiently. Approval for the study has been obtained from the medical ethics review board of Leiden Den Haag Delft, located at Leiden University Medical Center, the Netherlands (reference N20.170/ML/ml). Each participating hospital provided local approval for this study.

### Data Management

All sensitive data will be stored in encrypted password-protected databases (EUR Document Vault and Codific Document Vault [to save audio recordings during the study period]). Data will be entered by the study coordinator (LJP).

## Results

Ethical approval for the study was obtained in December 2020, and thereafter, until December 2021, each of the 12 participating hospitals obtained local approval for this study. The first clinician started with the individual SDM training program in May 2021. As of February 2022, we enrolled 30 clinicians, of whom 5 (17%) have completed the training program. The pace of participant inclusion in the study is increasing; therefore, study recruitment is planned to be finalized around July 2022. We plan to complete data analyses by December 2022.

A mixed cofunding was obtained from the participating clinicians themselves (voluntary contribution), from the Dutch OncoZon-Citrienfonds (a professional oncology network), CZ Health Care Insurer, and DSW-Phoenix Health Care Insurer.

The study results will be disseminated to partnering organizations, study participants, and organizations involved in the development of clinician education. The findings will be submitted to a peer-reviewed journal and presented at academic conferences.

## Discussion

### Principal Findings

We hypothesize that clinicians exposed to this intervention are more likely to adopt SDM behavior than clinicians who do not, resulting in decisions that better match the preferences and values of oncology patients. We expect that clinicians in the intervention group will increase their observed level of SDM after each part of the intervention. We also believe that the effect of the training program will be at least as large as the average increase that other interventions have shown [56]. Another possible effect is that patients may perceive greater involvement in the decision-making process and thereby experience a higher level of autonomy.

### Comparison With Prior Work

We have previously worked on designing effective interventions, including training, to help clinicians adopt SDM in daily practice [23,31,32]. The theory-based and blended approach builds on previous research and includes different types of clinicians, diagnoses, hospitals, and oncology decisions to stimulate generalizability [29]. This approach is grounded in the theory of deliberate practice [37]. Moreover, patient involvement is guaranteed in the design and implementation of this study. Therefore, the study is perceived to have global value and should engender considerable interest in the academic and clinical education fields.

### Strengths and Limitations

A strength of our approach is that it will be tailored to the context of individual clinicians and that it targets attitudes, knowledge, and skills of clinicians. The possible limitation of this protocol could be that participating clinicians may already have an inclination toward SDM, which can lead to selection bias. Therefore, we will try to invite clinical teams rather than individuals to participate in this study to include a group of clinicians with a wide range of SDM interests and skills. Another limitation is that the clinicians cannot be blinded to the intervention. This might encourage them to practice SDM apart from the intervention itself.

### Future Directions

This trial takes the next step in the pursuit of developing effective training methods for clinicians in the field of SDM. It will increase our knowledge about how effective and feasible the direct observation of audio-recorded health care encounters, followed by constructive feedback from a coach, can be. Principles of deliberate practice are used as the basis for the educational approach, which enables effective learning [37], and the intervention is substantiated by implementation theory (Meetinstrument Determinanten van Innovaties model) and a 4-step model for applying SDM during clinical consultations [21,48].

Our intervention incorporates important elements from the theory of deliberate practice, such as having a well-defined goal, motivation to improve, and providing feedback on real-life situations [37]. Nevertheless, in our delineated intervention, it is difficult to meet the hallmark of providing opportunities for repetition and gradual refinement of performance over time. Therefore, future studies should address this challenge.

### Conclusions

For most patients with cancer, multiple treatment options exist, and SDM is crucial to support them in making informed decisions that best fit their personal preferences. Clinicians play an important role in enhancing SDM implementation; however, SDM implementation remains challenging. This study will examine the effectiveness of an individual SDM training program for physicians. The results of this study will be disseminated through publication in an open-access journal to enable the uptake of this deliberate practice study in other fields of interest and through presentations. In the Netherlands, patient organizations, professional bodies, and health care insurers are involved in the project and are committed to using valuable results for daily practice. Although our educational intervention is a mixed set of interventions with several elements over a 10-week period, it is relatively short and labor intensive, with one-on-one feedback and coaching. For implementation, it is important to take this into account and continue to look for interventions that are applicable in daily (oncological) care as well as support a continuous learning process for clinicians.

## Abbreviations

CONSORT: Consolidated Standards of Reporting Trials
CPS: Control Preferences Scale
OPTION-5: Observing Patient Involvement–5
SDM: shared decision-making
SDM-Q-9: Shared Decision-Making Questionnaire–9
